# Optimized Eco-Friendly Foam Materials: A Study on the Effects of Sodium Alginate, Cellulose, and Activated Carbon

**DOI:** 10.3390/polym16172511

**Published:** 2024-09-04

**Authors:** Mehmet Emin Ergün, Rıfat Kurt, Ahmet Can, İsmail Özlüsoylu, Evren Ersoy Kalyoncu

**Affiliations:** 1 Akseki Vocational School, Alanya Alaaddin Keykubat University, Antalya 07630, Turkey; 2 Faculty of Forestry, Bartin University, Bartin 74100, Turkey; rkurt@bartin.edu.tr (R.K.); acan@bartin.edu.tr (A.C.); iozlusoylu@bartin.edu.tr (İ.Ö.); 3 Arsin Vocational School, Karadeniz Technical University, Trabzon 61900, Turkey; eersoy@ktu.edu.tr

**Keywords:** foam, sodium alginate, Box–Behnken design, activated carbon, cellulose

## Abstract

This study focuses on optimizing the physical and mechanical properties of foam materials produced with the addition of sodium alginate as the matrix, and cellulose and activated carbon as fillers. Foam materials, valued for their lightweight and insulation properties, are typically produced from synthetic polymers that pose environmental risks. To mitigate these concerns, this study investigates the potential of natural, biodegradable polymers. Various foam formulations were tested to evaluate their density, compression modulus, and thermal conductivity. The results indicated that an increase in activated carbon content enhanced thermal stability, as indicated by higher Ti% and Tmax% values. Additionally, a higher concentration of sodium alginate and activated carbon resulted in higher foam density and compressive modulus, while cellulose exhibited a more intricate role in the material’s behavior. In the optimal formula, where the sum of the component percentages totals 7.6%, the percentages (e.g., 0.5% sodium alginate, 5% cellulose, and 2.1% activated carbon) are calculated based on the weight/volume (*w*/*v*) ratio of each component in the water used to prepare the foam mixture. These results indicate that natural and biodegradable polymers can be used to develop high-performance, eco-friendly foam materials.

## 1. Introduction

Foam materials are multifunctional materials that offer advantages such as light weight, low thermal conductivity, high sound insulation, high impact absorption, and low cost [1]. These materials are widely used in various industries, such as in automotive, construction, packaging, textile, food, pharmaceutical, and biomedical applications. Among the most commonly used polymers in foam production are polyurethane, polystyrene, polyethylene, polypropylene, and polyvinyl chloride. Polyurethane is well known for its flexibility and cushioning properties, making it ideal for furniture and insulation [2]. Polystyrene is known for its excellent thermal insulation capabilities and is commonly used in packaging and disposable containers [3]. Polyethylene is known for being lightweight and having shock-absorbing properties, making it suitable for protective packaging. Polypropylene offers good chemical resistance and is typically utilized in automotive and consumer goods. Polyvinyl chloride is valued for its durability and fire resistance, commonly finding applications in construction and industrial settings [4,5]. However, these raw materials are synthetic polymers and generate wastes that are harmful to the environment [6]. Therefore, producing foam materials from natural and biodegradable polymers is an important research topic from both environmental and economic perspectives.

Biopolymers, like starch [7], chitosan [8], cellulose [9], guar gum [10], and sodium alginate [11], are used as matrix components in foam production. Sodium alginate, a natural polymer derived from seaweed, is an important additive that enhances the mechanical strength of foam materials by forming a cohesive matrix. Additionally, its biodegradability makes foam products more environmentally friendly and sustainable [12]. Cellulose is widely used as filler in composite foams due to its excellent mechanical properties, biodegradability, and availability. It enhances the strength and stiffness of the foam, contributing to a more robust and durable structure. Furthermore, the natural thermal insulation properties of cellulose make it an ideal candidate for applications where heat management is critical. Previous studies have demonstrated cellulose’s effectiveness in improving the mechanical and thermal performance of composite foams. Research highlights how cellulose fibers can create a network within the foam, enhancing its overall structural integrity [13,14].

Activated carbon, known for its high surface area, porosity, and excellent adsorption properties, is a key filler in composite foams. These features enhance thermal stability and adsorption capacity, making it useful for filtration and purification applications. The study showed that incorporating activated carbon into composite foams significantly improves thermal conductivity and stability, which are critical for applications requiring high thermal resistance [15].

Foams produced from bio-based matrixes and reinforced additives are high-performance and eco-friendly materials with a synergistic effect from each component. However, the optimal formulation and processing conditions of foam materials are not well established, and the effects of different factors on their physical and mechanical properties are not fully understood. Researchers have optimized the physical and mechanical properties of foam materials using the Box–Behnken design (BBD), a response surface methodology (RSM) technique that can efficiently explore the relationship between multiple factors and responses [16,17]. 

Optimizing foam properties with advanced designs like BBD has proven effective in producing high-performance materials. For instance, the previous study used BBD to optimize chitosan-based foams, showing its potential for refining material characteristics [17]. This study extends that work by applying similar techniques to sodium alginate-based foams, incorporating cellulose and activated carbon as novel fillers.

Cellulose and activated carbon make significant contributions to the mechanical and thermal properties of composite materials. Previous research has shown that natural fillers like cellulose can enhance the mechanical strength and thermal insulation of foams [18]. However, the effects of combining cellulose with other fillers, such as activated carbon, in a sodium alginate matrix have not been fully explored. The previous studies have highlighted activated carbon’s potential to improve thermal stability and conductivity in polymer composites, suggesting that its inclusion in bio-based foams could lead to the development of high-performance materials [19].

Achieving a consistent, homogeneous, and structurally stable foam can be challenging, especially when multiple fillers are added. The previous study examined the effects of filler content on the internal structure and mechanical properties of foams, revealing that excessive filler use can lead to clustering and uneven distribution, which can impact overall material performance [20]. This research aims to address these challenges by optimizing filler ratios and processing conditions to improve understanding of interactions within the foam matrix.

The primary purpose of developing this foam is to create an eco-friendly, high-performance material using the unique properties of natural and biodegradable components. Designed for applications requiring light weight and thermal insulation, such as construction, packaging, and thermal insulation, the foam uses natural alternatives like sodium alginate, cellulose, and activated carbon instead of synthetic polymers to ensure environmental sustainability. This study aims to optimize the physical, thermal, and mechanical properties of these foams. Component ratios were determined according to the Box–Behnken design, and foams were prepared. The physical properties, including density, compressive strength, and modulus, were measured and analyzed using regression models, interaction graphs, and response surfaces to determine the optimal conditions.

## 2. Materials and Methods

### 2.1. Materials

Cellulose was obtained from Konya Paper Industry (density: 0.53 g/cm^3^). Sodium alginate derived from brown algae, with a density of 1.0 g/mL at 25 °C and a molecular weight of 100,000 Daltons, was purchased from Sigma-Aldrich (St. Louis, MO, USA). Activated carbon and sodium dodecyl sulfate (SDS) were provided by Aromel Chemistry (Konya, Turkey). The density of the activated carbon was 2.14 g/cm^3^, with a BET surface area of 207 m^2^/g. It was produced from coconut shells through physical activation.

### 2.2. Production Process of Sodium Alginate-Based Foam

Foam materials were produced using a manufacturing technique known as ‘solvent-casting’, which involves molding a solution or suspension, followed by drying in an oven. Sodium alginate was used as the matrix material, while cellulose and activated carbon were utilized as fillers to produce the foam. The ratios of these components, as indicated in Table 1, correspond to their concentrations in the aqueous solution used during foam preparation. The production process began by dissolving sodium alginate in distilled water, with continuous stirring at 1400 rpm for 75 min to ensure a homogeneous solution. After the sodium alginate was fully dissolved, cellulose, known for enhancing mechanical strength, and activated carbon, valued for its thermal stability, were added to the solution as fillers. To ensure a homogeneous distribution, they were mixed into the solution at 850 rpm for 20 min.

**Figure 1:**
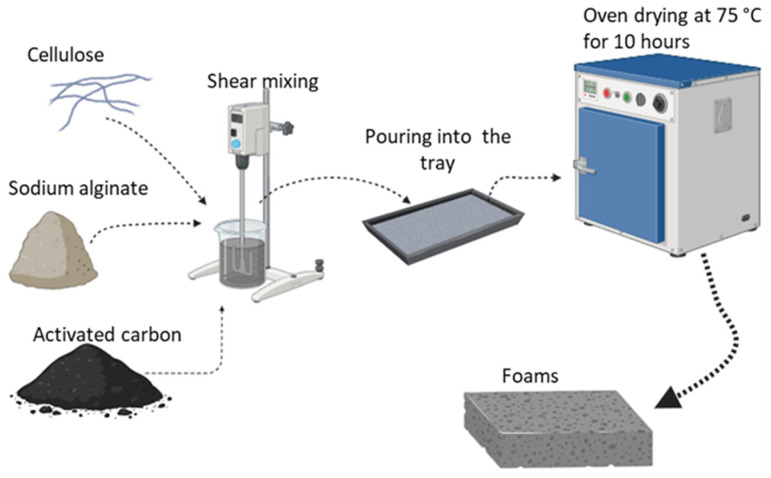
Production process of foam.

SDS was added to the mixture at a concentration of 8 g/L to generate the foam structure. The mixture was further stirred at 1100 rpm for 15 min, facilitating the formation of the foam through the action of the SDS as a foaming agent. The resulting foam mixture was then poured into molds and subjected to drying at 75 °C for 10 h, allowing the foam to solidify and retain its structure. The final foam product, conditioned for subsequent testing, demonstrates the combined effects of sodium alginate, cellulose, and activated carbon, with the concentrations of these components in the water-based mixture playing a crucial role in determining the foam’s physical and mechanical properties. The process for the production of the foam is shown in Figure 1.

### 2.3. Characterization of Sodium Alginate-Based Foam

All foams were conditioned for 24 h at 20 °C and 65% relative humidity before their physical and mechanical properties were measured. The density of the foamed material was determined with relevant standard [21]. Compression properties were evaluated using a universal testing machine (Instron 600 DX, Massachusetts, ABD) [22]. The dimensions of the foams were 10 × 10 × 2 cm^3^. The final strain was set at 25% of the original specimen height, and the compression rate was 8 mm/min. Thermal conductivity was measured using a heat flux meter (KEM QTM 500, Kyoto Electronics, Kyoto, Japan) [23]. The foam morphologies were examined using a JSM-7600F (Hefei, Anhui, China) scanning electron microscope (SEM). To enhance conductivity, gold was applied to the surfaces of the foams. The working voltage of the microscope was set to 15 kV for examining the microstructure images. Foams were subjected to thermal gravimetric (TG) studies at 600 °C using a Hitachi STA 7300 TG-DTG (Chiyoda, Tokyo, Japan) analyzer. Nitrogen passed through them at a rate of 50 mL/min and 10 °C/min for the duration of the experiment. Throughout the heating and pyrolysis process, the sample’s weight loss was monitored continually. Using the Rigaku SmartLab (Massachusetts, ABD) apparatus, X-ray diffraction patterns between 10° and 80° were examined. Density, compression, and thermal conductivity tests were conducted with six repetitions each.

### 2.4. Box–Behnken Design

Box–Behnken design (BBD) is a valuable tool in optimization processes, especially in the field of experimental design and response surface methodology. This design enables the simultaneous optimization of multiple variables while conducting a minimal number of experiments [24]. It is a three-level second-order spherical design where all points lie on a sphere, making it suitable for fitting second-order response surface models [25]. BBD has been instrumental in various fields, including chemistry, medicine, and environmental science, where the interactions between factors play a crucial role in determining outcomes [26,27].

The BBD can be integrated with response surface methodology (RSM) to assess the influence of various process variables on the desired response [17]. By employing these methods, researchers can systematically explore the parameter space, identify critical factors, and optimize processes to achieve desired outcomes [28].

In this study, three levels were determined for three parameters (sodium alginate, cellulose, and activated carbon) added to foam materials to optimize their physical, thermal, and mechanical properties (Table 1).

For each of the 15 combinations determined by the BBD design, three measurements were taken for the physical and mechanical properties at each level, and the averages were used for optimization. The BBD design is provided in Table 2.

## 3. Results and Discussion

**Figure 2:**
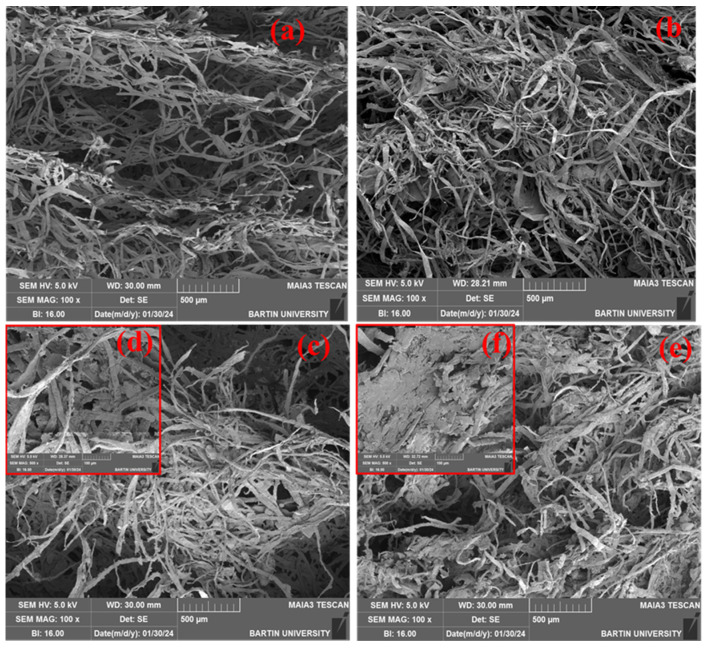
(**a**) Sample with 1% sodium alginate, 3% cellulose. (**b**) Sample with 1% sodium alginate, 3% cellulose, and 1% activated carbon. (**c**,**d**) Sample with 1% sodium alginate, 3% cellulose, and 3% activated carbon. (**e**,**f**) Sample with 1% sodium alginate, 3% cellulose, and 5% activated carbon.

In the first part of this study, SEM, XRD, and TGA analyses were conducted to observe the effect of activated carbon on the foam materials. The SEM images of foams showed a relatively loose and fibrous structure, characteristic of cellulose fibers embedded in a sodium alginate matrix, as shown in Figure 2.

The microstructures of samples containing different concentrations of sodium alginate, cellulose, and activated carbon are shown in Figure 2. The sample in Figure 2a contains 1% sodium alginate and 3% cellulose, and this sample exhibits the fibrous and porous structure typical of a foam material with relatively low alginate content. The cellulose fibers are well dispersed within the matrix. The absence of activated carbon results in a more open and less dense morphology. The sample in Figure 2b contains 1% sodium alginate, 3% cellulose, and 1% activated carbon. This image shows a denser network structure compared to Figure 2a, which may be attributed to the activated carbon addition. The presence of activated carbon enhanced the structural integrity, forming a more compact and interconnected network. The sample in Figure 2c,d contains 1% sodium alginate, 3% cellulose, and 3% activated carbon. These images demonstrate that adding more activated carbon significantly affects the microstructure, creating a more complex and dense network structure. Activated carbon particles contribute to the matrix by forming additional bonding points. The sample in Figure 2e,f contains 1% sodium alginate, 3% cellulose, and 5% activated carbon. In this study, the highest concentration of activated carbon resulted in noticeable clustering within the foam material. This clustering led to a significant increase in the material’s density and a reduction in its porosity. These results show that activated carbon significantly impacts the foam’s structure. It increases the material’s density and reduces the size and number of pores, which affects the foam’s overall mechanical and thermal properties.

Previous studies indicated that increasing the amount of polymer used as the matrix results in a denser and tighter internal structure [18,19]. On the other hand, an increase in filler content was found to reduce the porosity in the internal structure of the foam material [29]. Moreover, excessive addition of filler material, as seen in Figure 2f, was found to reduce bond formation due to clustering [30]. In previous studies, the morphological structure of the foams had a honeycomb shape [31,32]. Nevertheless, this study noted irregularities in the morphological structures of the foams. This is due to the oven drying technique and the macrosized cellulose that disturbs the cell structures and interstices. The previous studies found that foams produced from biopolymer derivatives such as chitosan [33], starch [34], and cellulose [9] had irregular and open cell pore structures. 

**Figure 3:**
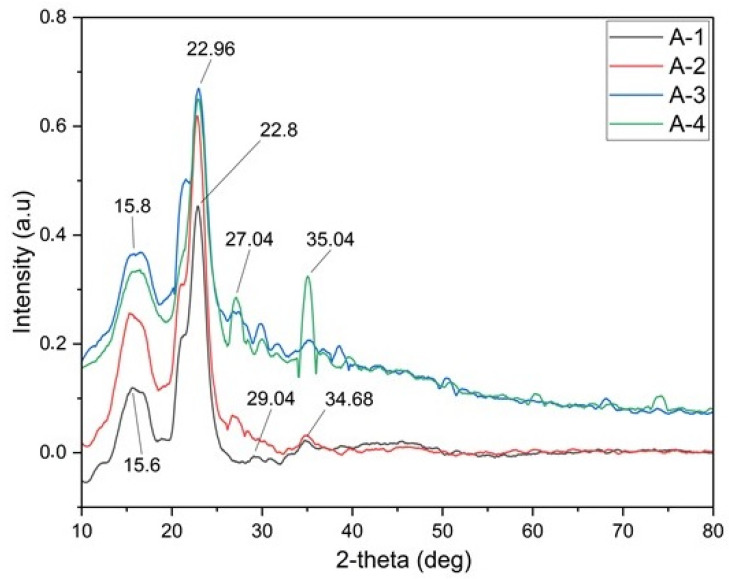
XRD analysis of foam (A-1: Sample with 1% sodium alginate, 3% cellulose. A-2: Sample with 1% sodium alginate, 3% cellulose, and 1% activated carbon. A-3: Sample with 1% sodium alginate, 3% cellulose, and 3% activated carbon. A-4: Sample with 1% sodium alginate, 3% cellulose, and 5% activated carbon).

The XRD results for the foam materials with different compositions are shown in Figure 3.

The XRD patterns of activated carbon-free foam material (A-1: 1% sodium alginate, 3% cellulose, and 1% activated carbon), and foam material containing increased amounts of activated carbon (A-2: 1% sodium alginate, 3% cellulose, and 1% activated carbon; A-3: 1% sodium alginate, 3% cellulose, and 1% activated carbon; A-4: 1% sodium alginate, 3% cellulose, and 1% activated carbon) are displayed in Figure 3. As can be seen in Figure 3 (A-1), the strong diffraction peaks of foam appeared at 15.6° and 22.8°, and are attributed to the sodium alginate and crystalline cellulose. A-1 foam material contains 1% sodium alginate and 3% cellulose. The strong crystal peak at 22.8° comes from cellulose. With the inclusion of active carbon, the shift in the peaks at 15.8° and 22.8° and the intensity of the peaks at points 27.04° and 35.04° increased. The planes are responsible for the considerable crystalline peaks that sodium alginate exhibits at 2θ = 31°, suggesting its notable crystallinity [35]. Sodium alginate exhibits two diffraction crystalline peaks at 13.5° (weak and broad) due to the hydrated crystalline Cellulose-I, 22°, and one peak at 39° due to the planes from the polyguluronate unit [36]. The typical structure is shown by the primary peaks in the cellulose diffraction peaks, which are located around 2θ = 16.5°, 22.5°, and 34.6° [37]. Activated carbon displays a straightforward XRD profile with two broad diffraction bands at 2θ = 23° and 43°, which correspond to the height and width of the crystallites and are typical of graphitic structures, and 57.7°, 37.9°, and 27.5° are the additional peaks at 2θ. The creation of carbon and graphite structures in the (002) and (100) planes is shown by peaks at 24.4° and 44.3°. Additionally, they exhibit a peak at 57.7°, indicating the existence of amorphous phases that are advantageous for forming adsorption sites due to their unevenly stacked carbon rings. The characteristic XRD peaks of sodium alginate, crystalline cellulose, and active carbon are superimposed over that of foam. Therefore, no new characteristic peaks of foam are observed in the XRD patterns, implying that the crystal structures of the foam composite are not affected by the direct impregnation treatment of active carbon. 

**Figure 4:**
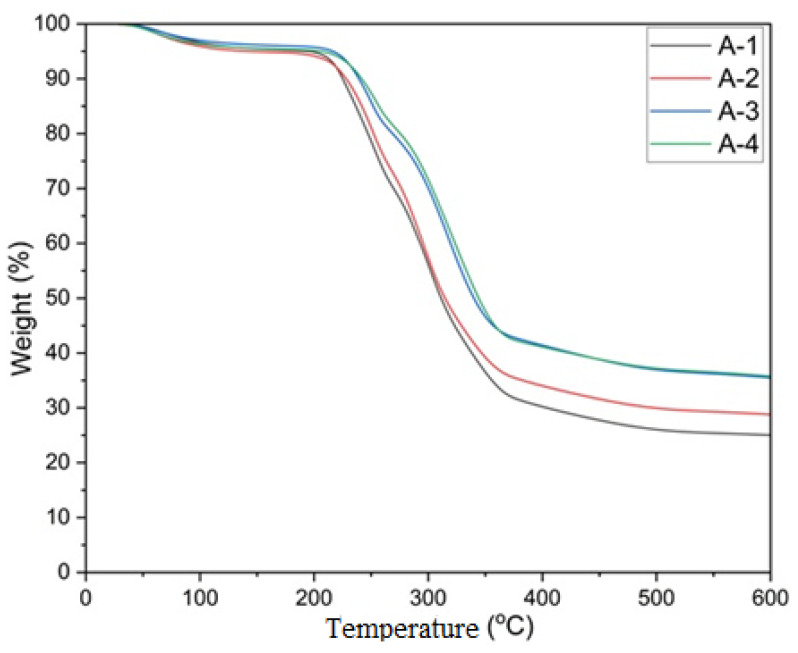
A-1: Sample with 1% sodium alginate, 3% cellulose, 0% activated carbon; A-2: sample with 1% sodium alginate, 3% cellulose, and 1% activated carbon; A-3: sample with 1% sodium alginate, 3% cellulose, and 3% activated carbon; A-4: sample with 1% sodium alginate, 3% cellulose, and 5% activated carbon.

The thermogravimetric analysis (TGA) conducted on the samples revealed significant insights into the thermal stability and degradation characteristics of the produced foam material. The weight-loss diagrams, depending on the temperature, of samples treated with varying compositions of sodium alginate, cellulose, and activated carbon are shown in Figure 4.

Weight-loss trends can be analyzed using TGA curves. Upon analyzing the TGA curves presented in Figure 4, it can be observed that the initial stage and the first instance of mass loss for all samples occur within the temperature range of 35–120 °C. This initial mass loss is mainly due to the dehydration of the samples. The second step at 190–400 °C is referred to as the decomposition (pyrolysis) step of hemicellulose and cellulose [38]. In terms of the similar trends in TGA curves in Figure 4, A-1 samples (1% sodium alginate, 3% cellulose, and 0% activated carbon) can be grouped with A-2 samples (1% sodium alginate and 3% cellulose) and A-3 samples with A-4 samples as two different groups. The rapid weight loss begins at around 200 °C and slows down at 350–400 °C. It was observed that the combustion process for the A-3 and A-4 samples concluded earlier than for the A-1 and A-2 samples. Compared with the A-1 and A-2 samples, the A-3 and A-4 samples were observed to have a higher char yield and lower mass loss within the same temperature ranges.

The combustion resistance properties were enhanced with the increasing ratio of activated carbon. According to the TGA curves of the samples, the rate of mass loss decreases with the increase in the ratio of activated carbon. In particular, the A-4 (1% sodium alginate, 3% cellulose, and 5% activated carbon) samples gained fewer flammable properties with a higher residual mass ratio, and exhibit higher thermal stability, thus obtaining the best result (Table 3).

Ti% values ranged from 225 °C to 241 °C, and Tmax% values ranged from 311 °C to 343 °C.

According to the data in Table 4, the maximum temperature difference requirement for reaching the maximum temperature point from the initial temperature for pyrolysis was obtained with samples A-3 (1% sodium alginate, 3% cellulose, and 3% activated carbon) and A-4 at 101 and 102 °C.

The highest remaining mass percentage after thermal degradation was 35.62% in sample A-4. The lowest remaining mass percentage was 24.96% in A-1 (1% sodium alginate, 3% cellulose, and 0% activated carbon). The samples with higher amounts of activated carbon exhibited better thermal stability compared to those without activated carbon. The best result was achieved with the A-4 sample, which exhibited the lowest mass loss.

The results indicate that the presence of activated carbon significantly enhances the thermal stability of the composites. This enhancement is evidenced by the progressive increase in both Ti% and Tmax% with higher concentrations of activated carbon. The initial and maximum decomposition temperatures were both higher in samples containing activated carbon, indicating better resistance to thermal degradation. Additionally, the remaining mass percentage after thermal degradation increased with the addition of activated carbon, suggesting that activated carbon contributes to a higher char yield during pyrolysis [8]. This higher residual mass is likely due to the formation of more thermally stable structures within the foam material.

This study investigates the effects of varying concentrations of sodium alginate, cellulose, and activated carbon on the density, compression modulus, and thermal conductivity of materials. The results are given in Table 4.

The produced foam is designed for applications that require lightweight and thermally insulating materials. The targeted applications include sectors such as automotive, construction, packaging, and thermal insulation applications, where such properties are critically important. Each additive in the foam composite serves a specific purpose. Added primarily as the base polymer matrix, sodium alginate provides the structural framework for the foam. It contributes to the overall cohesiveness and mechanical strength of the foam, helping to achieve a robust and stable structure. Cellulose was added to enhance the mechanical properties and thermal insulation of the foam. Its fibrous nature helps reinforce the foam matrix, contributing to density and stiffness. However, as discussed, its impact on thermal conductivity is complex, and it must be carefully balanced to avoid increasing heat transfer. The primary purpose of adding activated carbon was to improve the foam’s thermal stability and modify its thermal conductivity. Activated carbon also contributes to the mechanical properties, particularly in enhancing the compression modulus by providing additional structural support. However, its hydrophobic nature and tendency to aggregate must be managed to ensure uniform distribution and optimal performance.

The density of the composite materials ranged from 0.0313 g/cm^3^ to 0.093 g/cm^3^. The highest density was observed with a combination of 1.5% sodium alginate, 3% cellulose, and 5% activated carbon (0.093 g/cm^3^), while the lowest density was found with 0.5% sodium alginate, 3% cellulose, and 1% activated carbon (0.0313 g/cm^3^). Generally, increasing the concentration of sodium alginate or activated carbon tends to increase the density of the composite. Densities of 0.015–0.028.5 g/cm^3^ for microfibrillated cellulose foams, highlighting the potential for using low-density materials in such composites [32]. The density increase with higher sodium alginate and activated carbon concentrations is consistent with previous study [39]. 

The compression modulus values varied significantly, with a minimum of 0.0371 MPa and a maximum of 0.7057 MPa. The highest compression modulus was recorded for the sample containing 1.0% sodium alginate, 1% cellulose, and 5% activated carbon (0.7057 MPa). This indicates that the presence of higher amounts of activated carbon and sodium alginate enhances the composite’s stiffness. Conversely, the lowest compression modulus was found in the composite with 0.5% sodium alginate, 3% cellulose, and 1% activated carbon, suggesting that lower concentrations of these components result in less rigid composites. The compression modulus values between 1 and 97 MPa for alginate-based materials, emphasizing the influence of sodium alginate as a binder [40]. Similarly, compression modulus of activated carbon-reinforced chitosan foams values ranging from 0.214 to 0.394 MPa [8]. Previous studies have results similar to ours, showing enhanced stiffness with higher concentrations of sodium alginate and activated carbon. As the filler content increases, the density rises, which implies a reduction in the load applied to the foam material per unit mass. However, excessive addition of filler leads to clustering in the foam material matrix (Figure 2e,f), thereby diminishing its mechanical properties [17].

Thermal conductivity values ranged from 0.0275 W/mK to 0.0883 W/mK. The highest thermal conductivity was observed for the composite with 1.5% sodium alginate, 3% cellulose, and 5% activated carbon (0.0883 W/mK). The lowest thermal conductivity was recorded for the composite with 0.5% sodium alginate, 3% cellulose, and 1% activated carbon (0.0275 W/mK). The higher concentrations of activated carbon and sodium alginate contribute to higher thermal conductivity, while the influence of cellulose is less pronounced. The thermal conductivity of the foams is influenced by the composition and distribution of activated carbon [20]. The thermal conductivity values of different bio-based foams such as guar gum [10], sodium alginate/Al_2_O_3_ fiber [41], starch, and PVAc [42] varied between 0.027 W/mK and 0.074 W/mK. The foam’s porous microstructures were found to have favorable thermal insulation properties [43]. The gaps inside the foam structure are filled by air, which has low heat conductivity. The layered structure formed in foam materials restricts thermal conductivity due to the formation of voids in the pore walls [44].

Table 5 below summarizes the key properties, including density, compressive modulus, and thermal conductivity, of the eco-friendly foam from the current study and common synthetic foams such as polyurethane, polystyrene, and polyethylene. These properties are critical in determining the suitability of foam materials for various industrial applications, such as insulation, packaging, and cushioning.

This study demonstrates that the composition of sodium alginate, cellulose, and activated carbon significantly influences the physical, thermal, and mechanical properties of the composite materials. Increasing the amounts of sodium alginate and activated carbon generally enhances the density, compression modulus, and thermal conductivity of the composites. However, the effect of cellulose on these properties appears to be more complex, and further investigation is required to fully understand its role. 

### Statistical Analysis and Optimization

Analysis of variance (ANOVA) was employed to determine the individual interactions among the control factors. Initially, the assumption of normality, which is a critical prerequisite for the application of ANOVA, was investigated. The analysis determined that only the compression modulus values did not satisfy this condition. Therefore, a Box–Cox transformation (λ = −1: (1/y_i_) was applied to these data to meet the normality assumption. In the conducted variance analysis, the *p*-values for the independent variables were found to be less than 0.05 (with the exception of the cellulose variable for the compression modulus) (Table 6). This indicates that the variables in the model contribute significantly. According to the analysis results, the most effective factor for density, compression modulus, and thermal conductivity is the addition of activated carbon.

In the regression models established to predict density, compression modulus, and thermal conductivity, all independent variables, except for the cellulose variable for the compression modulus, were found to be significant at the 5% significance level. For density and thermal conductivity, high determination coefficients (R^2^) of 92.7% and 84.5%, respectively, were achieved, while an acceptable R^2^ value of 82.1% was obtained for the compression modulus. This indicates a good fit between the experimental data and the data predicted by the model. The regression models developed for prediction are provided in Equations (1)–(3). The mean squared error (MSE) values obtained for density, thermal conductivity, and compression modulus were 2.18 × 10⁻^5^, 5.31 × 10⁻^5^, and 7.53, respectively, with the compression modulus data being Box–Cox transformed. These low MSE values indicate that the predictive models for density and thermal conductivity exhibit high accuracy with minimal error, while the model for the compression modulus, despite demonstrating a relatively higher error, still falls within an acceptable range. Overall, the models are well suited for predicting the physical, thermal, and mechanical properties of the foam materials studied, confirming their reliability and effectiveness.

Density = 0.00339 + 0.01800 _SA_ + 0.004542 _C_ + 0.009292 _AC_ (1)

Compression Modulus= 31.3 − 16.9 _SA_ + 5.59 _C_ − 3.40 _AC_ + 6.30 _SA×SA_ − 0.965 _C×C_ − 0.278 _AC×AC_ − 1.99 _SA×C_+ 0.67 _SA×AC_ + 0.519 _C×AC_ (2)

Thermal Conductivity= −0.00294 + 0.01585 _SA_ + 0.00380 _C_ + 0.01026 _AC_ (3)

**Figure 5:**
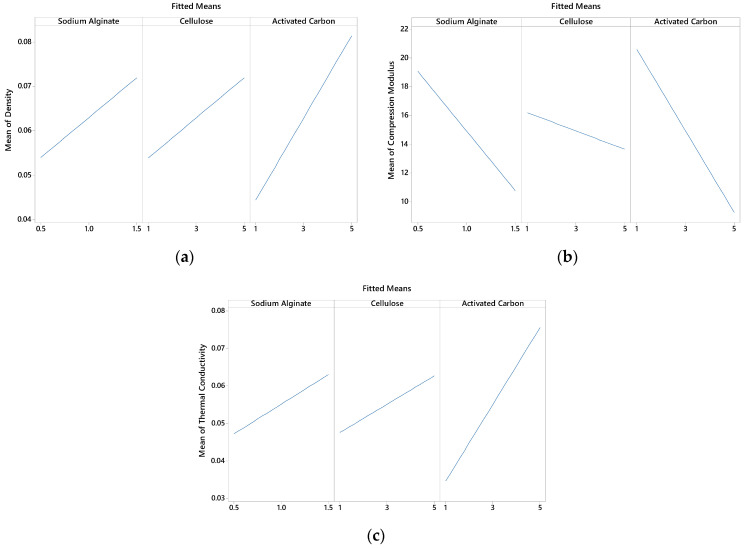
Main effect plots of physical and mechanical properties: (**a**) Density, (**b**) Compression modulus, (**c**) Thermal conductivity.

The main effects of using sodium alginate, cellulose, and activated carbon on the density, compressive modulus, and thermal conductivity of the foam material are shown in Figure 5. The use of sodium alginate and cellulose positively affected the density and thermal conductivity to an equal extent. In contrast, activated carbon significantly increased both the density and the thermal conductivity. All the materials used resulted in a decrease in the compressive modulus of the foam material. In Figure 5 of this study, the trends show that increasing the ratios of cellulose and activated carbon influences the density, compressive modulus, and thermal conductivity of the composite foam in specific ways.

Although cellulose is generally considered an excellent thermal insulator due to its porous and fibrous nature, the increase in thermal conductivity observed with higher cellulose content is indeed counterintuitive. This effect can be explained by the structural changes that occur within the composite when cellulose content is increased. When more cellulose is added, it can lead to a denser packing of fibers within the foam [9]. This densification reduces the number and size of air pockets, which are key contributors to low thermal conductivity [50]. Since air is a poor conductor of heat, reducing these air pockets can inadvertently increase the material’s ability to conduct heat [51]. As cellulose content increases, there is more direct contact between cellulose fibers. This creates more pathways for heat transfer through the solid material rather than through the insulating air gaps, resulting in higher thermal conductivity [52].

The composite’s overall thermal conductivity can be raised by using activated carbon, a material with moderate thermal conductivity and strong thermal stability. In comparison to the insulating qualities of the base polymer and cellulose, heat can pass through the conductive pathways that the particles of activated carbon produce as more is added, increasing the density of the foam [53].

The reduction in compression strength with the addition of either activated carbon or cellulose in the foam material, as observed in Figure 5, is indeed counterintuitive. Typically, adding micro- or nano-sized fillers to a composite material tends to enhance mechanical properties like compression strength. However, several factors in the context of these foam materials could explain why the opposite trend is observed. When activated carbon is added in higher amounts, especially without proper surface treatment or dispersion aids, it tends to aggregate. These aggregates can create weak points in the foam structure, leading to a reduction in mechanical strength rather than an enhancement [54]. Instead of reinforcing the matrix, these clusters can act as stress concentrators, making the material more prone to failure under compression [55]. Similarly, cellulose fibers, if not well dispersed, can clump together, leading to non-uniform distribution within the foam matrix. This can create areas with uneven mechanical properties, where some regions are overly stiff while others are weak, reducing the overall compression strength [56].

The mechanical reinforcement typically expected from fillers depends heavily on good interfacial bonding between the filler and the matrix [57]. If the interaction between the cellulose or activated carbon and the sodium alginate matrix is weak, the load transfer from the matrix to the filler is inefficient. This poor bonding can lead to a reduction in mechanical properties, as the fillers may not effectively bear or distribute the applied load during compression [58]. Sodium alginate and cellulose are hydrophilic, while activated carbon is more hydrophobic. The mismatch in their affinities can lead to poor bonding, particularly with activated carbon, further contributing to the reduction in compression strength [59].

Both cellulose and activated carbon, when added beyond a certain concentration, can disrupt the continuity and integrity of the polymer matrix. This disruption can manifest as voids, irregularities, or discontinuities within the foam structure, which weaken the material’s ability to resist compressive forces. Instead of providing reinforcement, excessive filler content can compromise the matrix, leading to a reduction in compression strength.

**Figure 6:**
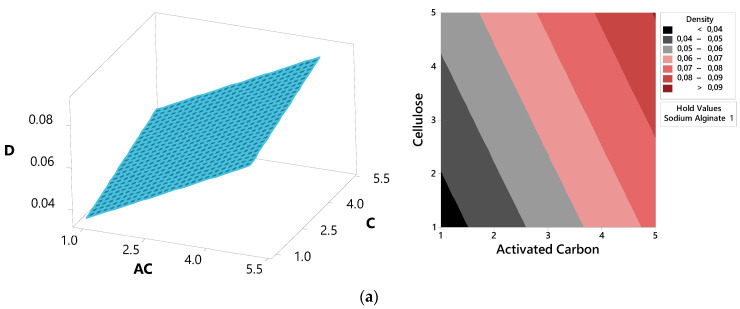

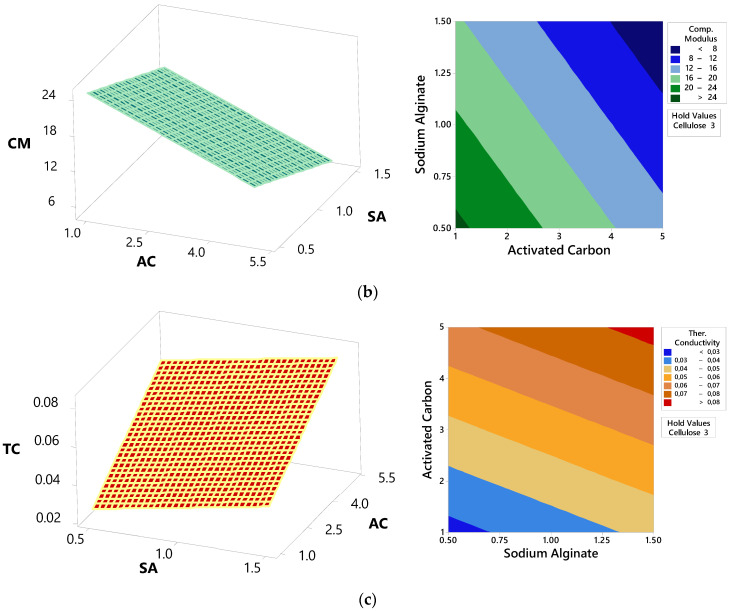
Response surface plots of physical and mechanical properties: (**a**) Effect of AC and C on D, (**b**) Effect of AC and SA on CM, (**c**) Effect of SA and AC on TC. (D: density, CM: compression modulus, TC: thermal conductivity, C: cellulose, SA: sodium alginate, AC: active carbon).

The variables with the most significant impact on the physical and mechanical properties were analyzed using response surface graphs (Figure 6). It was observed that the density increased proportionally with the increase in the usage of activated carbon and cellulose. The maximum density values were reached with 5% activated carbon and cellulose usage. Examination of the surface plot and contour plot graphs for the compressive modulus revealed that as the use of sodium alginate and activated carbon increased, the compressive modulus decreased, while thermal conductivity increased. The compressive modulus was at its highest, and thermal conductivity was at its lowest, with 0.5% alginate and 1% activated carbon usage.

**Figure 7:**
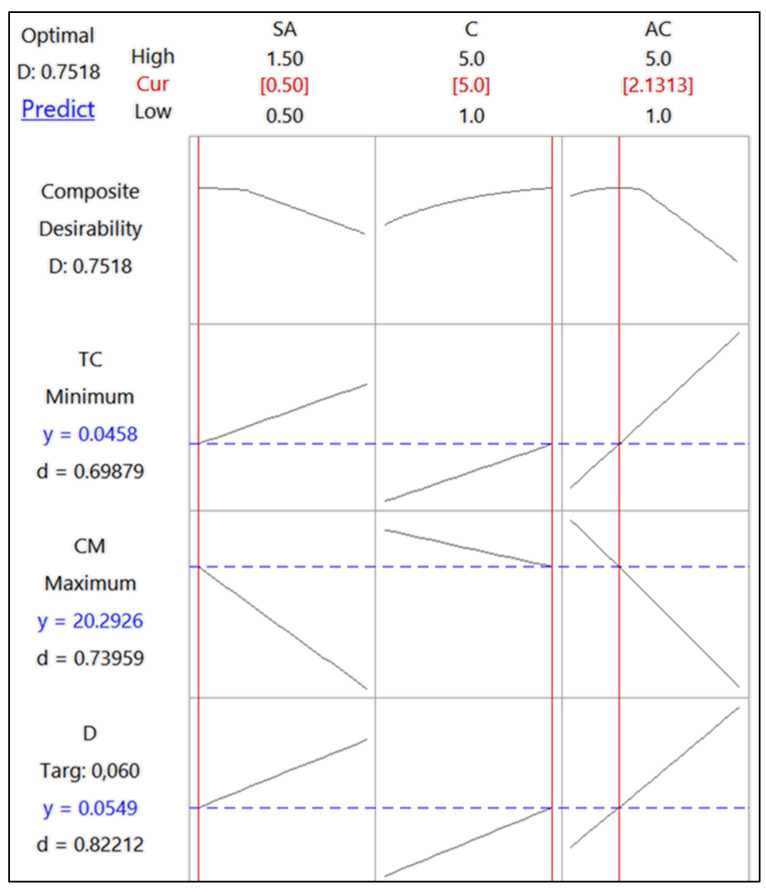
Optimization graph for factors and levels.

In our study, it was expected that the foam material produced would have minimum thermal conductivity, while the compressive modulus would be desired to be at its maximum. The ideal density was considered to be the target value, which is the average of our measurements, i.e., 0.06. The optimal conditions that met these targets were achieved with 0.5% sodium alginate, 5% cellulose, and 2.1% activated carbon (Figure 7). The desirability function (d) indicated an acceptable agreement between the predicted and experimental values. The percentages (e.g., 0.5% sodium alginate, 5% cellulose, and 2.1% activated carbon) are based on the weight/volume (*w*/*v*) of each component in the water used to create the foam mixture. This means that for every 100 mL of the solution, there is 0.5 g of sodium alginate, 5 g of cellulose, and 2.1 g of activated carbon. The sum of these percentages being less than 100% indicates that water is the primary component of the solution, acting as the medium in which these materials are dispersed before foam formation. After the drying process, water is removed, leaving behind the solid components that form the foam’s structure. The percentages refer to the solution used to produce the foam, and not the final composition of the dry foam. It is important to clarify that the 7.6% represents the total concentration of active materials in the preparation mixture. After drying, the concentration of these materials in the final foam is significantly higher, with water content mostly removed. The final properties of the foam are a result of the interactions between these components during and after the drying process, rather than their initial concentrations in the solution.

Previous studies have demonstrated the potential of various biopolymers, such as chitosan and starch, in the creation of foam materials. For instance, studies have shown that increasing the amount of biopolymer in the matrix leads to a denser internal structure, which is consistent with our observation that higher sodium alginate concentrations resulted in effects on density and mechanical properties [32,39].

The use of activated carbon in foam materials is another area where this study aligns with and expands upon prior research. Activated carbon has been previously reported to enhance thermal stability and mechanical strength due to its high surface area and adsorption properties [20,40]. Our results confirm these findings, showing that activated carbon significantly increased the thermal stability and compression modulus of the foam materials.

In contrast, the role of cellulose in foam materials is more nuanced. While some studies have reported that cellulose contributes to the structural integrity and stiffness of foams [8,9], our findings suggest that its effects are highly dependent on the concentration and its interaction with sodium alginate and activated carbon. This highlights a critical area for further investigation, particularly in understanding the balance required to maximize the benefits of cellulose without compromising other properties.

BBD, RSM, and other statistical analyses used in this study served as critical tools in optimizing the composition of the foam materials. While the model itself is not the primary focus of this study, its application enabled a systematic exploration of the parameter space, leading to the identification of optimal formulations that balance thermal and mechanical properties. The experimental results and their implications for the creation of sustainable materials, rather than the modeling procedure itself, are the true contribution of this study.

## 4. Conclusions

In this study, an environmentally friendly foam material developed by optimizing sodium alginate as the matrix and cellulose and activated carbon as fillers represents a significant step forward in foam material research. The incorporation of natural and biodegradable polymers into foam production has become a novel approach to reducing environmental impact. In this study, using the Box–Behnken design methodology, the physical and mechanical properties of the foams were successfully enhanced, demonstrating the potential of the optimization technique in creating high-performance materials. The results revealed that the foam materials produced with increased activated carbon exhibited improved thermal stability. Moreover, the synergistic effect of sodium alginate and activated carbon resulted in enhanced foam density and compression modulus. Although cellulose played multiple roles, its addition primarily enhanced the structural integrity and thermal insulation properties of the foams. This study contributes to the existing literature by elucidating the complex interactions between natural polymers and fillers in foam materials. The application of advanced experimental designs, such as the Box–Behnken design, underscores the efficacy of systematic optimization in tailoring foam properties. In the optimal formula, where the sum of the component percentages totals 7.6%, the percentages (e.g., 0.5% sodium alginate, 5% cellulose, and 2.1% activated carbon) are based on the weight/volume (*w*/*v*) of each component in the water used to create the foam mixture.

Overall, this study’s findings demonstrate that it is feasible to produce environmentally friendly foam materials with improved mechanical and physical properties by using natural and biodegradable polymers. Future research should focus on exploring the long-term stability and biodegradability of these materials under various environmental conditions, to further establish their viability as eco-friendly alternatives to conventional synthetic foams.

## Figures and Tables

**Table 1:** Parameters and levels.

Codes	Parameters	Levels		
		−1	0	1
A	Sodium Alginate (%)	0.5	1	1.5
B	Cellulose (%)	1	3	5
C	Activated Carbon (%)	1	3	5

**Table 2:** Box–Behnken experimental design of the composition of the foam.

Codes	Sodium Alginate (%)	Cellulose (%)	Activated Carbon (%)	Sodium Alginate (%)	Cellulose (%)	Activated Carbon (%)
1	−1	−1	0	0.5	1	3
2	1	−1	0	1.5	1	3
3	−1	1	0	0.5	5	3
4	1	1	0	1.5	5	3
5	−1	0	−1	0.5	3	1
6	1	0	−1	1.5	3	1
7	−1	0	1	0.5	3	5
8	1	0	1	1.5	3	5
9	0	−1	−1	1.0	1	1
10	0	1	−1	1.0	5	1
11	0	−1	1	1.0	1	5
12	0	1	1	1.0	5	5
13	0	0	0	1.0	3	3
14	0	0	0	1.0	3	3
15	0	0	0	1.0	3	3

**Table 3:** Ti% and Tmax% of thermal degradation and the mass loss (%) after thermogravimetric analysis.

Codes	Ti% (°C)	Tmax% (°C)	Char Yields (%)
A-1	225	311	24.96
A-2	227	314	28.67
A-3	238	339	35.33
A-4	241	343	35.62

**Table 4:** The density, compression modulus, and thermal conductivity test results for the foams.

Sodium Alginate (%)	Cellulose (%)	Activated Carbon (%)	Density (g/cm^3^)	Compression Modulus (MPa)	Thermal Conductivity W/mK
0.5	1	3	0.0400	0.0454	0.0356
1.5	1	3	0.0597	0.0601	0.0513
0.5	5	3	0.0687	0.0615	0.0595
1.5	5	3	0.0803	0.3455	0.0781
0.5	3	1	0.0313	0.0371	0.0275
1.5	3	1	0.0513	0.0545	0.0413
0.5	3	5	0.0723	0.0679	0.0731
1.5	3	5	0.0930	0.1136	0.0883
1.0	1	1	0.0430	0.0574	0.0412
1.0	5	1	0.0583	0.0557	0.0430
1.0	1	5	0.0797	0.7057	0.0738
1.0	5	5	0.0877	0.0975	0.0821
1.0	3	3	0.0593	0.0601	0.0438

**Table 5:** Performance characteristics of eco-friendly foam vs. synthetic foams.

Property	Eco-Friendly Foam (Current Study)	Polyurethane Foam	Polystyrene Foam	Polyethylene Foam
Density (g/cm^3^)	0.0313–0.0930	0.028 [45]	0.016–0.061 [46]	0.035–0.040 [47]
Compression Modulus (MPa)	0.0371–0.7057	0.42 [45]	0.6–3.1 [48]	0.7–4.20 [49]
Thermal Conductivity (W/mK)	0.0275–0.0883	0.031 [45]	0.033–0.040 [46]	0.041 [47]

**Table 6:** Analysis of variance results.

**Source**	**Density**				
	**df**	**SS**	**MS**	**F-Value**	***p*-Value**
Model	3	0.004071	0.001357	46.54	0.000
Linear	3	0.004071	0.001357	46.54	0.000
SA	1	0.000648	0.000648	22.23	0.001
C	1	0.000660	0.000660	22.64	0.001
AC	1	0.002763	0.002763	94.76	0.000
Error	11	0.000321	0.000029	-	-
Lack-of-Fit	9	0.000276	0.000031	1.37	0.491
Pure Error	2	0.000045	0.000022	-	-
Total	14	0.004391	-	-	-
**Source**	**Compression Modulus**				
	**df**	**SS**	**MS**	**F-Value**	***p*-Value**
Model	9	516.195	57.355	2.54	0.158
Linear	3	409.837	136.612	6.05	0.041
SA	1	138.464	138.464	6.13	0.056
C	1	12.841	12.841	0.57	0.485
AC	1	258.532	258.532	11.45	0.020
Square	3	71.387	23.796	1.05	0.446
SA × SA	1	9.150	9.150	0.41	0.552
C × C	1	55.010	55.010	2.44	0.179
AC × AC	1	4.580	4.580	0.20	0.671
2-Way Interaction	3	34.972	11.657	0.52	0.689
SA × C	1	15.910	15.910	0.70	0.439
SA × AC	1	1.789	1.789	0.08	0.790
C × AC	1	17.272	17.272	0.77	0.422
Error	5	112.868	22.574	-	-
Lack-of-Fit	3	106.983	35.661	12.12	0.077
Pure Error	2	5.884	2.942	-	-
Total	14	629.063	-	-	-
**Source**	**Thermal Conductivity**				
	**df**	**SS**	**MS**	**F-Value**	***p*-Value**
Model	3	0.004333	0.001444	19.91	0.000
Linear	3	0.004333	0.001444	19.91	0.000
SA	1	0.000502	0.000502	6.93	0.023
C	1	0.000462	0.000462	6.36	0.028
AC	1	0.003369	0.003369	46.44	0.000
Error	11	0.000798	0.000073	-	-
Lack-of-Fit	9	0.000789	0.000088	18.96	0.051
Pure Error	2	0.000009	0.000005	-	-
Total	14	0.005131	-	-	-

SA: sodium alginate, C: cellulose, AC: activated carbon.

## Data Availability

The data presented in this study are available on request from the corresponding author.
